# Tracking Differential Gene Expression in MRL/MpJ Versus C57BL/6 Anergic B Cells: Molecular Markers of Autoimmunity

**DOI:** 10.4137/bmi.s840

**Published:** 2008-06-10

**Authors:** Amy G. Clark, Katherine M. Mackin, Mary H. Foster

**Affiliations:** Departments of Medicine and Research Service, Duke University and Durham Veterans Affairs Medical Centers, Durham, North Carolina, U.S.A

**Keywords:** estrogen, autoimmunity, anergy, B cell, CD74, Ptpn22, Birc1f (Naip), Mef2c

## Abstract

**Background:**

Anergy is a key mechanism controlling expression of autoreactive B cells and a major site for failed regulation in autoimmune diseases. Yet the molecular basis for this differentiated cell state remains poorly understood. The current lack of well-characterized surface or molecular markers hinders the isolation of anergic cells for further study. Global gene profiling recently identified transcripts whose expression differentiates anergic from naïve B cells in model mouse systems. The objective of the current study was to evaluate the molecular and cellular processes that differentiate anergic cells that develop in the healthy C57BL/6 (B6) milieu from those that develop in the autoimmune-prone MRL/MpJ (MRL) background. This approach takes advantage of B6 and MRL mice bearing an anti-laminin Ig transgene with a well characterized anergic B cell phenotype.

**Results:**

Global gene expression was evaluated in purified transgenic B cells using Operon version 3.0 oligonucleotide microarray assaying >31,000 oligoprobes. Genes with a 2-fold expression difference in B6 as compared to MRL anergic B cells were identified. Expression of selected genes was confirmed using quantitative RT-PCR. This approach identified 43 probes corresponding to 37 characterized genes, including Ptpn22, CD74, Birc1f/Naip, and Ctla4, as differentially expressed in anergic B cells in the two strains. Gene Ontology classification identified differentiation, cell cycle, proliferation, development, apoptosis, and cell death as prominently represented ontology groups. Ingenuity Pathway Analysis identified two major networks incorporating 27 qualifying genes. Network 1 centers on beta-estradiol and TP53, and Network 2 encompasses RB1, p38 MAPK, and NFkB cell growth, proliferation, and cell cycle signaling pathways.

**Conclusion:**

Using microarray analysis we identified 37 characterized genes and two functional pathways engaged in maintenance of B cell anergy for which expression is distorted by underlying autoimmune genetic susceptibility. This approach identifes a new biological role for multiple genes and potential new therapeutic targets in autoimmunity.

## Introduction

Autoimmune diseases affect an estimated 6% of the population, causing significant morbidity and posing a major burden on the health care system (Nossal, 2001; Siatskas et al. 2006). Most current therapies such as steroids, cytotoxic agents, and broadly specific monoclonal antibodies rely on broad immunosuppression that is variably effective and carries the risk of infection, cancer, osteoporosis, sterility and other side effects. Novel interventions that act in a disease- or antigen-specific manner are urgently needed (Wolfraim, 2006), but their development requires a better understanding of the molecular basis for loss of immune tolerance that underlies disease pathogenesis.

Antigen-specific tolerance mechanisms that normally regulate autoreactive lymphocytes include cell deletion, receptor editing and anergy. Of these, B cell anergy is a particularly attractive target for therapeutic immune intervention *in vivo*, as suggested in preclinical models of lupus in which restoration of B cell expression of inhibitory FcγRIIB ameliorates nephritis (McGaha et al. 2005). Anergy is a final safeguard against autoreactivity when editing or deletion fails, and in autoimmune individuals is itself a major site of failed tolerance (Cambier et al. 2007; Conrad et al. 2007). The murine lupus-associated Ly108 susceptibility allele at the Sle1z/Sle1bz locus, for which there is an orthologous human locus, impairs B cell anergy as well as editing and deletion (Kumar et al. 2006). Anergy is a major control point in fulminant lupus nephritis in mouse models. B cell-specific inactivation of Cbl/Cbl-b leads to impaired anergy induction and severe immune complex glomerulonephritis (Kitaura et al. 2007). Yet little is known about the molecular blueprint of this important regulatory mechanism.

A major hurdle to therapeutically manipulating anergy in patients is the lack of information regarding anergy-specific markers or pathways to guide isolation of anergic B cells in man (Cambier et al. 2007). Unlike natural regulatory T cells, for which identification is aided by expression of the surface marker CD25+ and the intracellular foxp3 master regulator, B cell anergy is currently defined almost exclusively at the functional level. Although anergic cells are thought to comprise a significant proportion of splenic B cells, this tissue is not available for routine sampling in man. In contrast, anergic cells are estimated to comprise only 1%–5% of human peripheral B cells (Duty et al. 2006), which in turn comprise only approximately 5% of peripheral blood mononuclear cells. Alternative sources of anergic cells, such as *in vitro* tolerization, are limited due to the brief survival of unstimulated human B cells in culture.

This relative inaccessibility of human anergic cells led us to initiate studies using our LamH Ig transgenic mouse model system. Transgenic LamH Ig bind to the laminin alpha1-chain expressed in basement membranes in the kidney and other organs and targeted by autoantibodies in human lupus (Abrahamson et al. 2003; Amital et al. 2005; Amital et al. 2007). LamH Ig transgenic mice have a well-defined tolerance phenotype that includes anergy in peripheral B cells that escape central deletion (Brady et al. 2004; Foster et al. 2006; Hecox and Foster, 2004; Rudolph et al. 2002). Anergy is a state of cell differentiation that results from B cell receptor signaling in the absence of costimulation such that the cell fails to activate on subsequent engagement of antigen. Anergy in the LamH Tg model is characterized by impaired *in vitro* transgenic B cell proliferation and differentiation in response to Ig crosslinking and mitogen, compared to non-transgenic littermate B cells (Brady et al. 2004; Foster et al. 2006; Rudolph et al. 2002). This is accompanied by absence of serum transgenic autoantibodies, failure to recover autoreactive Tg monoclonal antibodies by fusion, shortened B cell life span, and, altered surface expression of activation and maturational markers. These Ig transgenic mice thus provide a reliable source of anergic B cells amenable to transcriptional interrogation.

Query of anergic cells by microarray permits measurement of relative levels of mRNA for thousands of unique genes and thus monitors a large number of putative regulatory pathways. This approach also allows screening for global changes in the absence of preconceived notions regarding gene function, in contrast to studies that focus on candidate molecules, pathways or genes on the basis of known roles in immune signaling or inflammation. Pioneering work by Glynne et al. used the hen egg lysozyme (HEL) Ig Tg model system and an early generation Affymetrix microarray spotted with ~4,000 murine genes. These investigators identifed a small subset of inhibitory genes differentially upregulated in anti-HEL anergic B cells, as compared to non-tolerant B cells, probed six hours after receptor stimulation (Glynne et al. 2000a; Glynne et al. 2000b).

To further explore the molecular basis of anergy involving a disease-relevant antigen, we used representational difference analysis (RDA) and the Operon v.3.0 oligoarray representing >31,000 elements to identify transcripts differentially regulated in anergic Tg as compared to naïve non-transgenic B cells (Clark et al. 2007). Results were initially mined for genes whose expression varied by >2-fold when comparing anergic to naïve cells in both the normal C57Bl/6 (B6) and the autoimmune-susceptible MRL/MpJ (MRL) strains, and selected results were validated by qPCR and Western blot. We identified approximately 60 genes deemed likely to include core regulatory molecules engaged in maintenance of anergy. This included genes encoding multifunctional proteins not previously implicated in B cell biology or tolerance but with roles in processes fundamental to the tolerance phenotype. Moreover, because their differential expression was common to B6 and autoimmune MRL anergic cells, these genes partially define minimal molecular requirements for anergy and allow us to establish a genetic profile of an anergic B cell.

Although both the normal B6 and the autoimmune MRL strains exhibited intact B cell tolerance in our anti-laminin Ig Tg, we hypothesized that subtle differences in the anergic phenotype of B cells between the strains may nonetheless contribute to the ultimate loss of tolerance leading to development of lupus in autoimmune MRL mice. In the current analysis of our gene expression database, we identified a distinct subset of genes that significantly differ in expression in autoimmune MRL as compared to B6 anergic cells. The majority of identified genes group into one of two functional networks known to regulate cell growth, signaling and apoptosis. Collectively, these studies provide novel insight into the biology of anergy and autoimmune genetic susceptibility. Moreover, identification of genes over or under expressed in anergic MRL B cells prone to tolerance failure suggests potential new biomarkers of autoimmune risk. Hopefully these findings will help guide efforts to identify the elusive anergic cell in man and to develop novel interventions to enhance tolerance in patients.

## Materials and Methods

### Animals

All studies and procedures were approved by the Duke University and the Durham Veterans Affairs Medical Center Animal Care and Use Committees. Cloning of the anti-laminin LamH CμH chain construct and thorough characterization of transgenic (Tg+) mice on both the standard B6 and the autoimmune MRL backgrounds have been described (Brady et al. 2004; Foster and Fitzsimons, 1998; Rudolph et al. 2002). Hemizygous Tg+ and non-transgenic (non-Tg) mice of either sex were reared under conventional conditions. B6 and MRL breeders were obtained from The Jackson Laboratory (Bar Harbor, ME, U.S.A.).

### B cell isolation

B cell isolation, RNA preparation, and microarray processing were performed as previously described (Clark et al. 2007). Briefly, splenocytes were isolated from LamH Tg+ and non-Tg control mice, matched for age and gender when possible. Splenocytes from each background were pooled by transgene status, and red cells removed by hypo-tonic lysis. Remaining cells were magnetically separated using a B cell negative selection kit (Miltenyi Biotech, Auburn, CA, U.S.A.) per the manufacturer’s protocol. The resulting B cell enriched population was then incubated with anti-B220/CD45R-PE fluorescently labeled antibody (Pharmingen, San Diego, CA, U.S.A.) and positive cells sorted and collected using a FACS DiVa flow cytometer (Becton Dickinson, Franklin Lakes, NJ, U.S.A.). Resulting B cell populations were generally >95% B220+. Cells were then either collected directly (t = 0) into buffer RLT from the Rneasy kit (Qiagen, Valencia, CA, U.S.A.) or were plated in proliferation media: RPMI 1640 supplemented with 10% heat-inactivated fetal bovine serum, 2 mM L-glutamine, 25 mM HEPES, 0.1 mM non-essential amino acids, 1 mM sodium pyruvate, 100 U/mL penicillin-streptomycin, 0.3% NaHCO_3_, and 2-mercaptoethanol (all supplements from Life Technologies, Carlsbad, CA, U.S.A.). Cultured cells were stimulated with 20 μg/mL anti-IgM F(ab′)_2_ (Pierce, Rockford, IL, U.S.A.) for 24 hours to crosslink B cell receptors simulating antigen contact. Following culture, viable cells were isolated using Lympholyte M (Cedarlane, Hornby, Ontario, Canada) and resuspended in buffer RLT (Qiagen). Lysed cells were homogenized using Qiashredder and stored at −80C until processing.

For qPCR studies: Splenocytes were harvested as described above. B cells were isolated by magnetic negative selection without subsequent FACS sorting. To enhance B cell purity in the Tg+ mice, 1/800th volume of additional anti-CD11b-biotin was spiked into the manufacturer’s antibody cocktail to more completely remove contaminating cells. Purified cells were collected directly (t = 0) into buffer RLT from the RNeasy kit (Qiagen).

### RNA isolation and amplification

RNA was extracted from purified B cells using the Rneasy mini- or midi-kit, depending on cell count, per the manufacturer’s instructions (Qiagen). On-column DNase digestion was simultaneously performed. For microarray studies, RNA was reverse transcribed and amplified for two rounds using the MessageAmp *in vitro* transcription kit (Ambion, Austin, TX, U.S.A.). For qPCR verification, RNA was reverse transcribed using random primers and the ImPromII kit (Promega, Madison, WI, U.S.A.) per the manufacturer’s instructions.

### Microarray

Anergic B cells were cohybridized against naïve B cells to microarray chips spotted with the Operon mouse v.3.0 oligo probe set, as previously described (Clark et al. 2007). For each biological replicate, dye reversed duplicate hybridizations were performed to eliminate dye bias. Labeling, hybridization, and scanning were performed by the Duke University Microarray Core facility as described (Clark et al. 2007) and can also be found at http://mgm.duke.edu/genome/dna_micro/core/spotted.htm. A total of 10 chips are included in this analysis, with dye-reversed replicates performed for each of 5 biological samples: one sample each from time 0 and following 24h of BCR crosslinking in each the B6 nonautoimmune background and the MRL autoimmune background, and an additional sample at 24h in the MRL background.

Data were imported into GeneSpringGX 7.3 analysis software, and dye swap and Lowess normalizations performed as appropriate. Data were initially filtered such that raw and control intensity values must be >100 for at least 7 of the 10 chips included, ensuring acceptable expression levels in at least 3 of the 4 time/strain categories. A volcano plot filter was then performed to identify genes whose expression ratio (anergic:naïve) differed by greater than 2-fold between B6 and MRL strains with a corrected p-value of <0.05.

### Quantitative PCR

Concentration of cDNA was estimated by spectrophotometry prior to qPCR and samples all brought to similar concentrations prior to analysis. Taqman primer/probe mixtures were ordered from Applied Biosystems (Foster City, CA, U.S.A.) for Ptpn22 (assay id Mm00501246_m1), Ap1gbp1 (assay id Mm00812653_m1), Birc1f/Naip (assay id Mm00783869_s1), and GAPDH (Mm99999915_g1). The primer/probe mixture was combined with cDNA and TaqMan Universal PCR Master Mix (Applied Biosystems) per the manufacturer’s protocol. The BioRad iCycler was used for the cycling program, with a 10 minute initial hold at 95C followed by 40 cycles of 15 seconds at 95C followed by 1 minute at 60C. Analysis was performed using iQ5 software (Biorad, Hercules, CA, U.S.A.). For each primer/probe set, a standard dilution curve was performed using purified clone DNA when available (Ap1gbp1) and non-Tg mouse B cell cDNA otherwise, with experimental cDNA starting gene quantities calculated by interpolation into this standard curve followed by normalization to GAPDH. Fold change in expression in anergic cells was calculated relative to similarly treated and concurrently isolated naïve B cells (from non-transgenic littermates).

### Gene Identification and Network Analysis

For each gene identified as differentially expressed by the above criteria, the probe sequence was submitted to BLAST (http://www.ncbi.nlm.nih.gov/BLAST/) to aquire the most up-to-date homologies and annotations. The Unigene ID and GenBank accession numbers of the best match for each gene, generally with 100% identity, were collected. These identifiers were used to submit the data list to both DAVID, or Database for Annotation, Visualization and Integrated Discovery (Dennis et al. 2003), to acquire complete ontological (GO) information, and to Ingenuity Pathway Analysis software (Ingenuity Systems, Redwood City, CA, U.S.A.) to assimilate network involvement of these genes.

## Results

### Expression differences in B6 versus MRL anergic B cells

**Figure 1:**
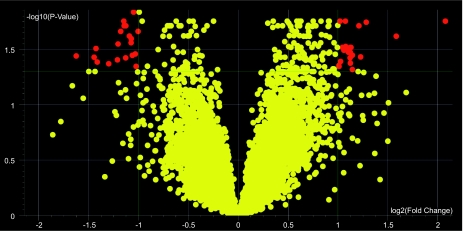
A volcano plot was used to visualize and identify genes, shown in red, whose expression ratio between anergic and naïve B cells differed between MRL and B6 strains. Cutoff criteria for differential expression in this analysis is defined as a minimum fold change of 2.0, plotted on the X axis, with a multiple testing corrected p-value (plotted on the Y axis) of less than 0.05.

**Figure 2:**
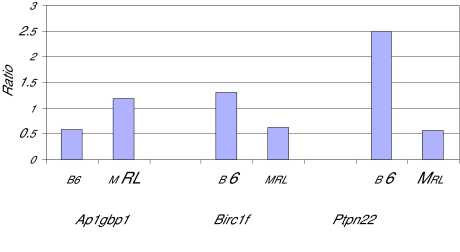
Quantitative PCR verification of three genes identified by microarray as differentially regulated in anergic B cells in the B6 versus MRL backgrounds. Unstimulated B cells were isolated from transgenic and nontransgenic mice in both backgrounds and quantified by RT-PCR using pre-designed TaqMan probe/primer sets. Relative copy number was calculated after normalization to expression levels of GAPDH in each sample. Shown is the ratio of anergic:naive transcript levels in each background. Genes shown are Ptpn22, protein tyrosine phosphatase non-receptor type 22; Birc1f baculoviral IAP repeat-containing 1f, also known as Naip6, or neuronal apoptosis inhibitory protein 6; and Ap1gbp1, AP1 gamma binding protein 1.

Using oligonucleotide microarray assaying >31,000 oligoprobes, we initially compared genes expressed in murine anergic and naïve B cells, derived from either autoimmune MRL or healthy B6 mice, using Genespring software to generate an anergic:naïve expression ratio, where <1.0 indicates relative increased expression in naïve B cells, and >1.0 indicates relative greater expression in anergic cells (not shown). We next generated a volcano plot to analyze expression ratios for each gene in B6 as compared to the MRL strain, using a 2-fold difference and p < 0.05 p-value cutoff (Fig. 1). This approach identified 43 probes with 2-fold or greater differential expression in B6 anergic as compared to MRL anergic B cells, standardized to naïve B cell expression. These probes corresponded to 37 characterized genes, listed in Table 1. Two genes, CD74 and IgK-V1, were identified by two different probes, whereas four additional probe sequences (Table 1, footnote) had no 100% homologous described murine sequences in GenBank as of February, 2008. Proteins encoded by the genes and additional common names for selected genes, where available, are included in Table 1. Within our cut-off criteria, gene expression differences between strains ranged from 2- to 3-fold. Differential expression was independently verified for three of these genes using real-time qPCR (Fig. 2). For Ptpn22, the expression difference determined by qPCR exceeded 4-fold between strains.

To explore the biological significance of gene expression patterns, we analyzed the Gene Ontology (GO) classification using the DAVID Bioinformatics Resource, courtesy of the National Institute of Allergy and Infectious Diseases. Approximately half of the identified genes cluster into six prominently represented ontology groups, with multiple genes falling into more than one category (Table 1): differentiation, cell cycle, proliferation, development, apoptosis and cell death. Other functional groups represented more than once in the dataset include signal transduction, transcriptional regulation, cytoskeletal organization, nucleotide binding, alternative splicing, phosphorylation/dephosphorylation, hydrolase, and immune response. Expression in B cells has been described for a subset of the identified genes, although for many genes the function in B cells is poorly explored. The role of some of these genes in B cell biology will be discussed below.

### Analysis of gene regulatory networks

**Figure 3:**
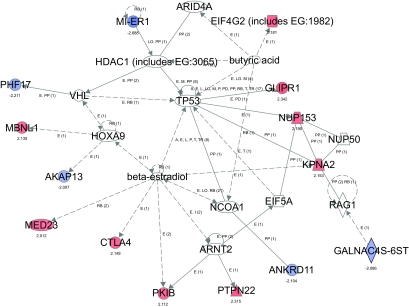

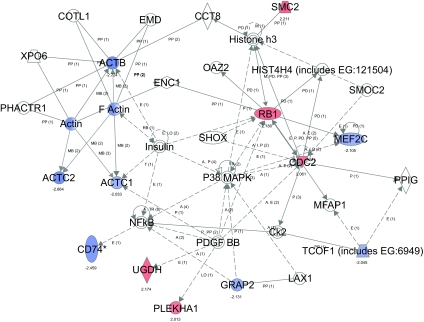
Network analysis of genes differentially expressed in B6 versus MRL anergic B cells. Ingenuity software generated two major networks (labeled 1 and 2) interconnecting multiple array-identified genes (shown in solid blue for expression lower in B6 than MRL and in red for higher expression in B6). The numbers displayed below array-identified genes indicate relative fold-change in B6 anergic cells. The networks summarize interactions between functional pathways and the genes that are altered in expression.

To further understand the molecular processes engaged in anergic cells and disrupted in autoimmunity, Ingenuity Pathway Analysis software was used to identify regulatory networks enriched amongst the genes showing differential expression. This software utilizes expert (manually) modeled information derived from the full text of articles published in top peer-reviewed journals to track molecular interactions. Of the 37 initially identified and previously characterized genes, 32 were available within the software database for network analysis. Two major networks were identified (Fig. 3), that incorporated 14 and 12, respectively, non-overlapping qualifying genes. Network 1 centers on beta-estradiol, and Network 2 encompasses cell growth, proliferation and cell cycle signaling pathways.

These networks integrate members of multiple functional subpathways, as suggested by GO analysis and including apoptosis, mitogen-activated protein kinase (MAPK) and nuclear factor kappaB (NFkB) signaling, and actin cytoskeletal interactions. Major hubs of Network 1 show that genes with differential expression interact with or are regulated via beta-estradiol, homeobox A9 (HoxA9) and Arnt2. Three additional qualifying genes, Eif4G2, Mier1 and Glipr1, are incorporated within the beta-estradiol regulated network when Network 1 is extended to include interactions with the TP53 gene that encodes transcriptional regulator protein p53. Hubs of Network 2 indicate crosstalk and regulation through Rb1, Cdc2, P38/MAPK, NFkB, Pdgf-BB, the actin family, histone H3, and insulin. Although not shown in Figure 3, the two major networks are interconnected by several interactions, including crosstalk between TP53 and Rb1 gene products, and Pkib regulation of various MAPK, extracellular signal-regulated kinase (ERK) and protein kinase A (PKA) pathways. Some signaling components are omitted from the displayed networks for the sake of clarity.

## Conclusion

We used oligoarray probes and an autoantibody transgenic model to identify 43 probes representing 37 characterized genes that are differentially expressed by 2-fold or more in B cells anergized within the context of the nonautommune B6 as compared to lupus-prone MRL genetic backgrounds. To our knowledge this is the first global gene expression analysis of anergic B cells derived from strains differentially susceptible to systemic lupus. The data provide evidence that autoimmune susceptibility alters B cell anergy at the level of gene expression. Identified genes include Ptpn22, previously identified as important in human autoimmunity and a potential therapeutic target, and CD74, a key modulator of B cell maturation and survival. Our findings are the first to link these molecules to B cell anergy. Gene Ontology categories development, differentiation, cell cycle, proliferation, apoptosis, and cell death were prominently represented. These categories also include cell functions known to be affected by previously described abnormalities associated with genetic or environmental susceptibility to systemic autoimmunity. Network analysis using Ingenuity Pathway Analysis software revealed that at least 26 of the identified genes are interconnected by signaling and regulatory pathways well described in immune and inflammatory responses, with nodes at TP53, Rb1, NFkB, P38/MAPK, Pdgf-BB and Cdc2. Network analysis also unmasked beta-estradiol at the center of one module interacting with 11 qualifying genes. Collectively, these results suggest an important role for these genes and signaling networks in the biology of anergy and autoimmune genetic susceptibility.

Several differentially expressed genes deserve emphasis. Our array findings are the first to link Ptpn22 to anergy. Ptpn22, tyrosine phosphatase non-receptor type 22, is a hematopoietic cell-specific intracellular phosphatase and known negative regulator of antigen receptor signaling in lymphocytes (Cloutier and Veillette, 1999; Hasegawa et al. 1999). Our arrays showed that an oligonucleotide corresponding to Ptpn22, and the mouse orthologue Ptpn8, is upregulated in anergic as compared to naïve B cells in both B6 and MRL mice, but with a two-fold higher level in B6 cells. Ptpn22 encodes a proline-, glutamic acid-, serine- and threonine-rich (PEST)-enriched tyrosine phosphatase, Lyp (human) or PEP (mouse) (Cohen et al. 1999; Wicker et al. 2005). In man, the C1858T (R620W) allelic variant of Ptpn22 has been linked to multiple human autoimmune disorders that have a prominent humoral component, including SLE, rheumatoid arthritis, type 1 diabetes, and autoimmune thyroiditis (Begovich et al. 2004; Bottini et al. 2004; Criswell et al. 2005; Gregersen et al. 2006; Kyogoku et al. 2004; Lauwerys and Wakeland 2005; Smyth et al. 2004). The role of Ptpn22/Ptpn8 as a susceptibility gene in mouse autoimmune diseases is less well characterized, although gene variants have been linked to type 1 diabetes in NOD mice (Wicker et al. 2005). Much of current knowledge of Ptpn22 function derives from studies of T cells and PEP, which implicate PEP in dephosphorylation of Src family kinases. The human R620W allele was recently determined to encode enhanced inhibitory function of the phosphatase, and individuals with the allele have dampened B and T cell receptor signaling (Rieck et al. 2007; Vang et al. 2005). These biological consequences were unanticipated, and fuel speculation that autoimmunity is caused by an aberrant Lyp-induced raised signaling threshold in lymphocytes of affected individuals, such that self-antigen contact fails to trigger tolerance induction. Our array results suggest that in healthy B6 mice an upregulated Ptpn22/Ptpn8 may be one mechanism for maintaining nonresponsiveness in anergic cells, perhaps via the raised signaling threshold, and that this mechanism is abrogated in autoimmune MRL mice. In cells from the MRL strain, the relative expression of Ptpn22 in the anergic:naïve subsets varied depending on the method of detection (array and qPCR), suggesting an influence of gene polymorphisms, splice variants, or other factors; protein expression studies should help resolve this point. The role of Ptpn22 in tolerance needs further exploration in anticipation of clinical application of recently developed Lyp inhibitors (Yu et al. 2007). It is notable that Pep-deficient mice fail to spontaneously develop overt autoimmunity, although they do develop large germinal centers, increased Ig concentrations, enhanced TCR-mediated signaling, and increased numbers of splenic CD4+ and CD8+ effector/memory T cells (Hasegawa et al. 2004).

B6 and MRL anergic B cells also vary by 2-fold or more in their expression of CD74, a nonpolymorphic Type II transmembrane protein, MHC Class II chaperone, and dormant transcription factor known to influence B cell biology. The relative expression of CD74 in anergic versus naïve B cells is inverse in the two mouse strains: CD74 is relatively downregulated in anergic cells from B6 mice, but upregulated in anergic cells from autoimmune-prone MRL mice. Although CD74 has not previously been linked to anergy, tolerance or autoimmunity, it has several biologic functions consistent with such a role. CD74 is crucial for B cell maturation, as shown in mice deficient in CD74 (Shachar et al. 1995; Viville et al. 1993). Recent studies reveal additional roles for CD74 in promoting B cell differentiation, proliferation and survival (Benlagha et al. 2004; Gore et al. 2008; Lantner et al. 2007; Shachar and Flavell, 1996; Starlets et al. 2006), and suggest a therapeutic role for antagonistic anti-CD74 antibodies in diseases marked by B cell overactivity (Stein et al. 2007).

The natural ligand for CD74 in B cells is unclear, although the CD74 extracellular domain is known to have high-affinity for the proinflammatory macrophage migration-inhibitory factor, MIF. CD74 is implicated in several MIF-mediated cell responses in mouse macrophages and the human Raji B cell line (Leng et al. 2003), including ERK, Syk and PI3K/Akt phosphorylation. Subsequent intramembrane proteolytic cleavage of CD74 triggers its nuclear translocation to induce transcription via NF-kB p65/RelA homodimer and its coactivator, TAF(II)105 (Becker-Herman et al. 2005; Matza et al. 2002a; Matza et al. 2002b; Matza et al. 2001; Starlets et al. 2006). MIF-induced CD74 signaling in B cells requires formation of a complex with CD44 (Gore et al. 2008), a molecule shown to be increased in both B6 and MRL unstimulated anergic B cells (microarray data not shown; cell surface expression in B6 mice is described in Brady et al. 2004).

Two additional genes identified as differentially expressed in anergic B6 as compared to MRL B cells have functions either known or anticipated to modulate autoimmunity. Cytotoxic T lymphocyte antigen-4, also known as Ctla-4 or CD152, a transmembrane protein primarilyknown as a major inhibitor of T cell responses (Teft et al. 2006), is upregulated in B6 relative to MRL anergic B cells in our array studies. This suggests a negative regulatory role for B cell CTLA-4, consistent with recent results in bone marrow chimeric mice in which CTLA-4 deficiency was restricted to B cells (Quandt et al. 2007). Birc1f, or baculoviral IAP repeat-containing 1f, also known as neuronal apoptosis inhibitory protein or Naip, is inversely regulated in B6 and MRL anergic B cells. Birc1f is a Nod-like and pathogen pattern recognition receptor and one of the four main defining groups of the newly described CATERPILLER family of proteins implicated in immunity and inflammation (Ting et al. 2006). Although its function in B cells is unknown, it is notable that Birc1f is downregulated during endotoxin-induced maturation of human dendritic cells from an immature tolerogenic to an immunogenic mature phenotype (Matsunaga et al. 2002). By analogy, Birc1f down-regulation in MRL anergic B cells may promote enhanced cell survival and antigen presenting functions that in turn promote autoimmunity. For multiple additional genes in our panel, including Mbnl1, Phf17, Prei4, Loh11cr2a, Glipr1, Tmbim1, Smc2, Tcof1, Ugdh, transcriptional activator Med23/Sur2, Actg2, Esco1, Ddx46, Ap1gbp1, Ankrd11, Actc1, Aldoart2, and Cept1, little is currently known about their biology in B cells.

In addition to identifying individual genes that may act independently to modify anergy and autoimmunity, our array studies identified molecular interactions and signaling networks that may have an additive or synergistic impact on tolerance and autommunity. Perhaps most notable, network analysis revealed an unexpected and central role for beta-estradiol influences in the maintenance of B cell anergy. Estradiol interacts with at least 11 genes, or their protein products, identified by our array analysis. Estrogens have long been suspected of promoting systemic autoimmunity due to the striking female predominance, including a female-to-male ratio of 10:1 in human SLE and earlier disease onset in females in some murine lupus models, such as (NZBxNZW)F1 mice (Andrews et al. 1978). Moreover, administration of exogenous estrogen has been shown to induce autoantibodies in non-autoimmune mice and accelerate disease in lupus-prone mice of both sexes, whereas ovariectomy or administration of testosterone to female lupus mice ameliorates disease (Apelgren et al. 1996; Melez et al. 1980; Roubinian et al. 1978; Steinberg et al. 1980; Verthelyi and Ahmed 1994; Verthelyi and Ahmed 1998).

The underlying cellular and molecular mechanisms for hormone linked effects are still not well understood. B cells express both estrogen receptor (ER)-alpha and ER–beta, although relative levels vary during cell development (Grimaldi et al. 2002). Exposure of healthy mice to elevated levels of estradiol alters expression of molecules known to regulate B cell receptor signaling (Grimaldi et al. 2002) and suppresses B cell lymphopoiesis (Kincade et al. 2000). Non-autoimmune mice rendered genetically deficient in estrogen or its receptors develop expanded B lymphoid and myeloid cell pools (Shim et al. 2003), plasmacytosis (Shim et al. 2004a), and autoimmunity (Shim et al. 2004a; Shim et al. 2004b), including lupus-like immune complex glomerulonephritis (Shim et al. 2004a), whereas induction of ER-alpha deficiency in (NZBxNZW)F1 lupus mice leads to amelioration of autoimmune disease (Bynote et al. 2008). These findings are difficult to reconcile, and suggest complex hormonal effects on B cell biology and immunity.

Novel insight into the role of estrogen in immune tolerance was provided in an elegant series of studies from Diamond and colleagues showing that estrogen blocks deletion of anti-DNA B cells at the transitional cell stage (Bynoe et al. 2000; Grimaldi et al. 2002; Grimaldi et al. 2006; Grimaldi et al. 2001; Venkatesh et al. 2006). Prolonged administration of 17beta-estradiol to Balb/c mice bearing the R4A-gamma2b H chain Ig transgene induced CD4 T cell-independent autoantibody production, bcl-2 expression, and expansion of unmutated high affinity anti-DNA B cells that are normally tolerized by anergy and deletion (Bynoe et al. 2000; Grimaldi et al. 2001). Estrogen-induced elevation in levels of B cell activating factor, or BAFF (also known as B-lymphocyte stimulator or BLyS) has been implicated (Grimaldi et al. 2006).

Our array results indicate that estrogen directly or indirectly interacts with molecules differentially engaged in maintenance of B cell anergy in B6 as compared to lupus-prone MRL mice. Estrogen-linked genes relatively upregulated in B6 as compared to MRL anergic cells include Ptpn22 and Ctla-4 (see above), Mbnl1, Med23, Nup153, Kpna2, and Pkib. Little is known about B cell functions of many of these genes. Nup153 is a nucleoporin and an autoantibody target in lupus (Ball and Ullman, 2005; Enarson et al. 2004). The B cell functions of Kpna2, or karyopherin alpha 2, also known as importin alpha 1, a protein engaged in nuclear transport and modulation of cell proliferation (Kussel and Frasch, 1995), are yet to be elucidated. Estradiol interacts with the protein kinase inhibitor, Pkib, both directly and via Arnt2, the arylhydrocarbon receptor nuclear translocator 2 that encodes a basic helix-loop-helix/PAS (Per-Arnt-Sim) transcription factor. Our array results suggest that the relative decrease in Pkib’s negative regulatory functions in MRL anergic B cells may contribute to autoimmunity.

Estradiol may regulate some genes in our panel indirectly through its effects on transcription, phosphorylation, and localization of the tumor suppressor protein p53 (Bond and Levine, 2007), encoded by gene TP53 (Fig. 3). Protein p53 is a nuclear phosphoprotein and DNA-binding transcription factor that regulates the cell cycle and apoptosis. Dysregulation of p53 pathways can induce lupus-like autoimmunity (Salvador et al. 2002), and p53 is itself a target of lupus autoanti-bodies (Herkel et al. 2001). TP53 interacts with three additional array-identified genes whose functions in B cells are not yet characterized: Glipr1 and Eif4G2, genes relatively upregulated in B6 versus MRL anergic B cells, and Mier1, which is relatively downregulated in B6 cells. Glioma pathogenesis-related protein (Glipr1) is a novel p53 target gene with proapoptotic activities in tumor cells (Li et al. 2008), whereas the translation initiation factor Eif4G2 has effects on the cell cycle (Lee and McCormick, 2006). Mesoderm induction early response gene 1, or Mier1, functions as a transcriptional repressor of a number of genes, including targets of transcription factor Sp1, through recruitment of histone deacetylase 1 (Hdac1), a major regulator of chromatin structure and gene expression (Ding et al. 2003). Although Mier1 expression and Hdac1 functions in B cells are not well characterized, Hdac1 is ubiquitously expressed in lymphoid tissues, and Hdac inhibitors have been used to induce cell cycle arrest and downregulate inflammatory cytokines and nephritis in MRL/lpr lupus mice (Mishra et al. 2003).

In contrast to the genes described above, several estrogen-interacting genes are relatively upregulated in lupus-prone MRL, as compared to B6, anergic B cells. These include Akap13, GalNAc4S-6ST and Ankrd11. Akap13, or PKA anchoring protein 13, also known as breast cancer nuclear receptor auxiliary factor or BRX, is a scaffold protein with Rho guanine nucleotide exchange factor activity that integrates extracellular signaling through Rho familyGTPase, PKA, p38/MAPK and NFkB. These pathways enhance nuclear hormone receptor signaling and transcriptional activity of ER-alpha and beta (Driggers et al. 2001; Kino et al. 2006; Rubino et al. 1998; Shibolet et al. 2007). GalNAc4S-6ST, also known as B cell Rag-associated protein or BRAG, is reported to enhance BCR signaling (Verkoczy et al. 2000). Relative upregulation of Akap13 and GalNAc4S-6ST in MRL B cells may promote cell activation or bypass of anergy. Little is currently known about the biological functions of ankyrin repeat domain 11, or Ankrd11.

The mechanisms through which estrogen interacts with genes and proteins in B cells remains poorly understood. A role in processes maintaining anergy is consistent with estrogen’s actions on non-lymphoid cells: estrogen regulates growth, proliferation, survival, and apoptosis through the classical ER-alpha and ER-beta and their functional splice variants, as well as through putative nonclassical membrane-localized receptors, of which G-protein-coupled receptor (GPR)30 is best characterized. ER-alpha and ER-beta regulate unique gene expression patterns, mediate isoform-specific and often opposing effects on cell functions, and cross-regulate each other’s expression and actions, through two distinct signaling pathways. In the delayed nuclear (formerly, genomic) pathway, ER acts as a ligand-dependent transcription factor that binds target gene promoters, whereas a rapid cell membrane-initated (nongenomic) pathway activates MAPK and PI3K/Akt signaling (Moriarty et al. 2006). Better characterization of the pathways regulated by estradiol during selection of autoreactive B cells should facilitate understanding of the sexual dimorphism of lupus susceptibility and lead to new therapeutic targets in SLE.

In addition to the estradiol and TP53 network, our array analysis linked an additional 13 genes, including CD74 discussed in detail above, Cdc2, Mef2c, Grap2 and Plekha, to a second interconnecting network with major nodes at molecules known to be key in B cell receptor signaling (NFkB, MAPK) and proliferation (retinoblastoma gene, Rb1). For several of these genes, their relative expression level in B6 as compared to MRL anergic cells runs counter to what might be anticipated based on current limited understanding of their biology. Rb1 protein was previously linked to B cell tolerance in a murine lymphoma model (Joseph et al. 1995). Rb1 hypophosphorylation and inhibition are associated with cell cycle and growth arrest (Bilancio et al. 2006; Joseph et al. 1995). It is notable that B cell survival factor BAFF/BlyS, a therapeutic target in SLE, promotes B cell Rb1 synthesis and phosphorylation (Huang et al. 2004). The significance of relative Rb1 upregulation in our B6 anergic cells is unclear. RB1 crossregulates Cdc2 and Mef2c, genes relatively up and down regulated, respectively, in B6 as compared to MRL anergic B cells. Impaired function of cell cycle regulator and cyclin-dependent kinase Cdc2 has been linked to T cell tolerance (Li et al. 2006). Mef2c, or myocyte enhancer factor 2, belongs to a small family of calcineurin-regulated calcium-dependent transcriptional regulators of lymphocyte signaling, cytokine production and apoptosis (Esau et al. 2001; Youn et al. 1999). Mef2c was recently determined to be critical to B cell receptor-induced cell proliferation and survival (Wilker et al. 2008). GRB2-related adaptor protein 2, or Grap2, and pleckstrin homology domain containing family A (phosphoinositide binding specific) member 1, or Plekha/TAPP1, genes down-and upregulated, respectively in B6 anergic cells, were recently determined to modulate B cell receptor signaling (Allam and Marshall, 2005; Garrett-Sinha et al. 2005). Neither Mef2c, Grap2, or Plekha was previously linked to tolerance or autoimmunity.

Although much remains to be done to elucidate the role of many of these genes in B cell anergy, this work represents a first attempt to identify genes and proteins that underlie failure of B cell tolerance in autoimmunity. Ongoing studies are using this information in an attempt both to identify cellular markers of anergy, with the hopes of translating these findings to description and isolation of human anergic cells, and to identify potential clinical targets for therapeutic intervention to prevent or ameliorate disease.

## Figures and Tables

**Table 1:** 

**Table 1A.** Gene ontology of differentially expressed transcripts identifed by microarray				
Gene symbol	Description	Ratio anergic: naïve		Ontologic categories
		B6	MRL	
**Genes increased 2-fold or more in B6 relative to MRL anergic B cells.**				
Loh11cr2a	Loss of hyeterozygosity 11	2.85	0.95	Cyc
Prei4	Preimplantation protein 4	1.48	0.61	Hydrolase, carbohydrate metabolism
Glipr1	GLI pathogenesis-related 1	2.36	1.01	Apop, Sig Trans
Ptpn22	Protein tyrosine phosphatase non-receptor type 22	3.61	1.56	Apop, Growth, Diff, Sig Trans, Dev, Phos
Tmbim1	Transmembrane BAX inhibitor motif containing 1	2.32	1.05	Alternative splicing
Smc2	Structural maintenance of chrom.2	1.06	0.48	Cyc, Nuc, chromosome organization
Nup153	Nucleoporin 153	1.14	0.52	Nuc, protein binding, transporter
Rb1	Retinoblastoma 1	1.28	0.59	Cyc, Phos, Trans reg, Diff, Prolif, Dev, Apop, Death
Eif4g2	Eukaryotic translation initiation factor 4 gamma 2	1.10	0.50	Nuc, Cyc., death, transcription initiation
EG622976		1.45	0.67	none
Ugdh	UDP-glucose dehydrogenase	1.32	0.61	Dev, electron transport
Kpna2	karyopherin (importin) alpha 2	1.25	0.58	DNA recombination regulation
Ctla4	Cytotoxic T-lymph-assoc. protein 4	1.96	0.91	Immune response
Mbnl1	Muscleblind-like 1	1.85	0.87	Diff, RNA binding
Pkib	Protein kinase inhibitor beta	1.24	0.59	Diff, kinase inhibitor
Birc1f	Baculoviral IAP repeat-containing 1f or Naip6	2.03	0.96	Apop, death
Cept1	choline/ethanolamine phosphor-transferase 1	1.52	0.73	Lipid metabolism
Cdc2a	Cell division cycle 2 homolog A	1.51	0.73	Apop, Nuc, Cyc, Phos, mitosis, DNA damage response
Plekha1	Pleckstrin homology domain containing family A	1.22	0.61	lipid binding, alternative splicing
Med23	Mediator subunit SUR2	1.20	0.59	Trans reg, metastasis suppressor
**Table 1B.**				
**Gene symbol**	**Description**	**Ratio anergic: naïve**		**Ontologic categories**
		**B6**	**MRL**	
**Genes decreased 2-fold or more in B6 relative to MRL anergic B cells.**				
Akap13	A kinase (PRKA) anchor protein 13 or BRX	0.91	1.83	Sig Trans
Actc1	Actin, alpha, cardiac	0.93	1.90	Dev, Nuc, Cytoskeletal organization
Tcof1	Treacher Collins Franceschetti syndrome 1 homolog	0.69	1.42	Prolif, Phos, transport, transcription
Aldoart2	Aldolase 1, A isoform, retrogene 2	0.91	1.89	none
GALNAC4S-6ST	B cell Rag-Associated protein	0.81	1.70	Catalytic activity
Ankrd11	Ankyrin repeat domain 11	1.05	2.20	Nuc
Mef2c	Myocyte enhancer factor 2C	0.91	1.92	Apop, Diff, Phos, Trans reg, Dev
Grap2	GRB2-related adaptor protein 2	0.65	1.38	Sig Trans, cell communication
Ddx46	DEAD box polypeptide 46	0.77	1.68	Nuc, RNA binding/splicing, hydrolase
CD74	CD74 antigen	0.84	1.85	Prolif, Apop, Sig Trans, Immune response
Phf17	PHD finger protein 17, Jade1	0.81	1.79	Apop, Dev, cell growth
Ap1gbp1	Ap1 gamma binding protein 1	0.67	1.50	ion binding, protein localization
Esco1	Establishment of cohesion 1	1.16	2.63	Cyc, alternative splicing, metal/ion binding
Igk-V1	Ig kappa V1	2.06	4.77	Immune Response
Actg2	Actin, gamma 2, smooth muscle	0.86	2.29	Nuc, Cytoskeletal organization, Dev.
Mier1	Mesoderm induct. early response-1	0.80	2.14	Nuc, Sig Trans, Phos., Trans reg
Actb	Similar to cytoskeletal beta actin	0.94	2.56	Nuc, Cytoskeletal organization

1. Genes represented by two probes are CD74 and Igk-V1. Expression ratios for one probe for each gene is shown in the table. Ratios for the second probe were 0.72 (B6) and 1.77 (MRL) for CD74, and 1.38 (B6) and 3.18 (MRL) for Igk-V1. GALNAC4S-6ST corresponds to Unigene 4631426J05Rik. Four genes or probes were omitted from the table due to absence of a known 100% homologous murine coding sequence. Operon identifiers for the omitted probes are M300017393, M300013500, M300019467, and M300013958.

2. Common GO categories: Development = Dev, Differentiation = Diff, Apoptosis = Apop, Proliferation = Prolif, Cell Cycle = Cyc, Cell Death = Death, Phosphorylation/Dephosphorylation = Phos, Transcriptional regulation = Trans reg, Signal Transduction = Sig Trans, Nucleotide binding = Nuc.

3. The ratios shown in the tables are the mean normalized values (across 4–6 chips) for the gene expression in anergic B cells relative to naive B cells for each strain. This is based on two-color competitive hybridization on each chip, pairing anergic B cell aRNA and naive B cell aRNA from the same mouse strain. The ratio of expression for each gene for each strain was generated, and these ratios were then compared between strains.
